# *N*,*N*′-(Propane-1,3-di­yl)bis­(2-amino­benzamide)

**DOI:** 10.1107/S160053681300888X

**Published:** 2013-04-10

**Authors:** Jagannatha Swamy Sreedasyam, Jyothi Sunkari, Shashank Kundha, Raghava Rao Gundapaneni

**Affiliations:** a Department of Chemistry, Kakatiya University, Warangal 506 009, India

## Abstract

The title compound, C_17_H_20_N_4_O_2_, was prepared by the reaction between 1,3-di­amino­propane and isatoic anhydride in water. The carbonyl O atoms are involved in intra­molecular hydrogen bonding with the amine group and inter­molecular hydrogen bonding with an amide H atom of an adjacent mol­ecule. In the crystal, pairs of N—H⋯O hydrogen bonds link mol­ecules into inversion dimers and further N—H⋯O hydrogen bonds link the dimers into ladder-like chains along the *a* axis.

## Related literature

For related mol­ecules and syntheses, see: Clark & Wagner (1944 ▶); Swamy & Kumar (1996 ▶); Swamy *et al.* (2003 ▶, 2004 ▶).
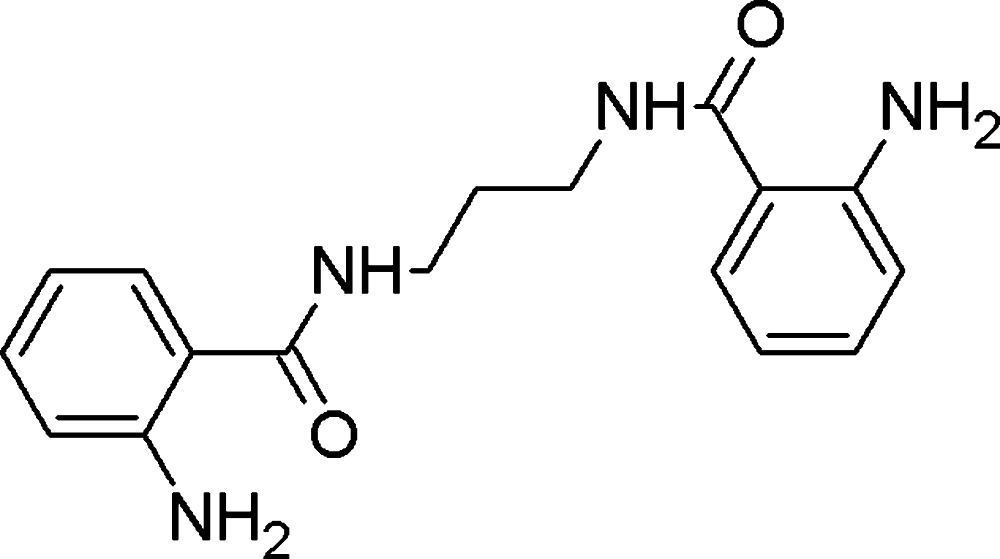


## Experimental

### 

#### Crystal data

C_17_H_20_N_4_O_2_
*M* *_r_* = 312.37
Triclinic, 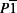

*a* = 5.6590 (7) Å
*b* = 9.8279 (12) Å
*c* = 14.6732 (18) Å
α = 95.258 (2)°
β = 95.263 (2)°
γ = 98.888 (2)°
*V* = 798.21 (17) Å^3^
*Z* = 2
Mo *K*α radiation
μ = 0.09 mm^−1^
*T* = 298 K
0.32 × 0.22 × 0.20 mm

#### Data collection

Bruker APEXII CCD diffractometer
Absorption correction: multi-scan (*SADABS*; Bruker, 2001 ▶) *T*_min_ = 0.972, *T*_max_ = 0.983
6168 measured reflections
3048 independent reflections
2539 reflections with *I* > 2σ(*I*)
*R*_int_ = 0.026

#### Refinement

*R*[*F* ^2^ > 2σ(*F* ^2^)] = 0.047
*wR*(*F* ^2^) = 0.124
*S* = 1.06
3048 reflections
232 parameters
H atoms treated by a mixture of independent and constrained refinement
Δρ_max_ = 0.16 e Å^−3^
Δρ_min_ = −0.17 e Å^−3^

### 

Data collection: *APEX2* (Bruker, 2001 ▶); cell refinement: *SAINT* (Bruker, 2001 ▶); data reduction: *SAINT*; program(s) used to solve structure: *SHELXS97* (Sheldrick, 2008 ▶); program(s) used to refine structure: *SHELXL97* (Sheldrick, 2008 ▶); molecular graphics: *ORTEP-3* for Windows (Farrugia, 2012 ▶) and *Mercury* (Macrae *et al.*, 2008 ▶); software used to prepare material for publication: *WinGX* (Farrugia, 2012 ▶).

## Supplementary Material

Crystal structure: contains datablock(s) I, global. DOI: 10.1107/S160053681300888X/rk2394sup1.cif

Structure factors: contains datablock(s) I. DOI: 10.1107/S160053681300888X/rk2394Isup2.hkl

Supplementary material file. DOI: 10.1107/S160053681300888X/rk2394Isup3.cml

Additional supplementary materials:  crystallographic information; 3D view; checkCIF report

## Figures and Tables

**Table 1:** Hydrogen-bond geometry (Å, °)

*D*—H⋯*A*	*D*—H	H⋯*A*	*D*⋯*A*	*D*—H⋯*A*
N4—H1*N*4⋯O2	0.87 (2)	2.10 (2)	2.711 (3)	126.6 (18)
N1—H2*N*1⋯O1	0.89 (2)	2.20 (2)	2.851 (2)	130.2 (19)
N3—H3*N*3⋯O1^i^	0.902 (19)	2.055 (19)	2.9286 (16)	162.8 (15)
N2—H2*N*2⋯O2^ii^	0.882 (17)	1.942 (18)	2.7872 (17)	160.3 (16)

Symmetry codes: (i) 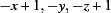; (ii) 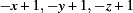.

**Fig. 1.**
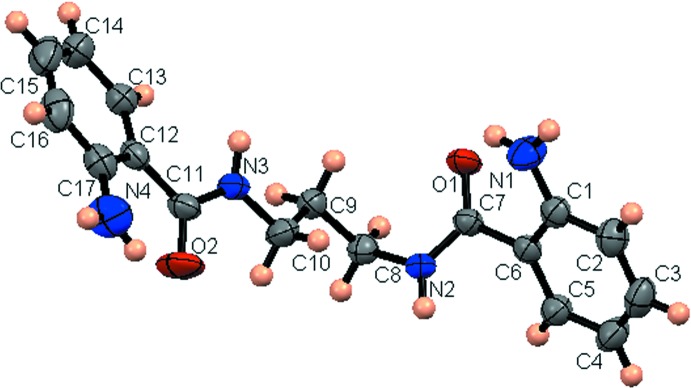
The molecular structure of title compound with the atom numbering scheme. Displacement ellipsoids are drawn at the 50% probability level. H atoms are presented as a small spheres of arbitrary radius.

**Fig. 2.**
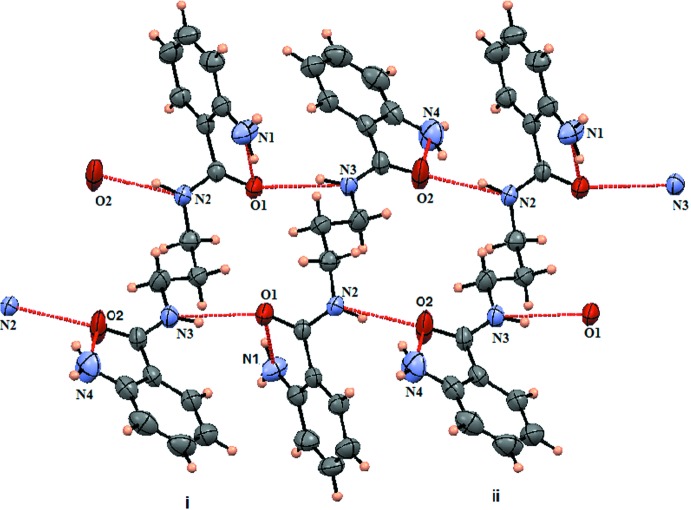
The formation of centrosymmetrical dimers by classical H bonds (N—H···O type) in crystal structure of title compound - dotted lines. Intramolecular H bonds are indicated by dotted lines too. Symmetry codes: (i) -x+1, -y, -z+1; (ii) -x+1, -y+1, -z+1.

## supplementary crystallographic information

### Comment

The reactions of isatoic anhydride with amines have been very interesting and yield molecules with amine and amide functional groups (Clark *et al.*, 1944). We have adopted the reaction of isatoic anhydride with different diamines to obtain a good number of organic molecules having two amine and two amide groups (Swamy & Kumar, 1996; Swamy *et al.*, 2003; 2004). The *N*,*N*'–propyl–bis–(2–aminobenzamide) I was prepared by the reaction between isatoic anhydride and 1,3–diaminopropane (Swamy & Kumar, 1996). We recrystallized I from water/methanol mixture to obtain block shaped single crystals. Herein we report the molecular and crystal structures of the title compound. The molecular structure of I is shown in Fig. 1 and the packing diagram with inter– and intra–molecular H–bonds resulting in supramolecular assembly and the formation of ladder like chain is shown in Fig. 2. The carbonyl oxygen is involved in the formation of intramolecular H–bond with amine hydrogen and intermolecular H–bond with amide hydrogen of the adjacent molecule. These intermolecular H–bonds lead to the formation of ladder like chain as shown in Fig. 2.

### Experimental

The title compound was synthesized by adding 1.15 mg of 1,3–diamminopropane (12.25 mmol) in 15 ml water to 4.0 mg of isatoic anhydride (24.5 mmol) with continuous stirring. Effervescence was observed while warming the reaction mixture on water bath that ceased after one hour. Microcrystalline solid product was obtained on allowing the mixture to stand overnight. The product was purified and recrystallized from methanol / water mixture to obtain block shaped crystals, m.p. 341 K.

### Refinement

The H atoms based on C atoms were positioned geometrically with C—H = 0.93Å for aromatic H and C—H = 0.97Å for methylene H, refined using a riding model with *U*_iso_(H) = 1.2*U*_eq_(C). The H atoms based on N atoms were found from difference Fourier map and refined freely.

### Crystal data

**Table:**

C_17_H_20_N_4_O_2_	*Z* = 2
*M**_r_* = 312.37	*F*(000) = 332
Triclinic, *P*1	*D*_x_ = 1.300 Mg m^−^^3^
Hall symbol: -P 1	Melting point: 341 K
*a* = 5.6590 (7) Å	Mo *K*α radiation, λ = 0.71073 Å
*b* = 9.8279 (12) Å	Cell parameters from 3048 reflections
*c* = 14.6732 (18) Å	θ = 1.4–25.9°
α = 95.258 (2)°	µ = 0.09 mm^−^^1^
β = 95.263 (2)°	*T* = 298 K
γ = 98.888 (2)°	Block, colourless
*V* = 798.21 (17) Å^3^	0.32 × 0.22 × 0.20 mm

### Data collection

**Table:**

Bruker APEXII CCD diffractometer	3048 independent reflections
Radiation source: fine-focus sealed tube	2539 reflections with *I* > 2σ(*I*)
Graphite monochromator	*R*_int_ = 0.026
φ and ω scans	θ_max_ = 25.9°, θ_min_ = 1.4°
Absorption correction: multi-scan (*SADABS*; Bruker, 2001)	*h* = −6→6
*T*_min_ = 0.972, *T*_max_ = 0.983	*k* = −11→11
6168 measured reflections	*l* = −17→17

### Refinement

**Table:**

Refinement on *F*^2^	Primary atom site location: structure-invariant direct methods
Least-squares matrix: full	Secondary atom site location: difference Fourier map
*R*[*F*^2^ > 2σ(*F*^2^)] = 0.047	Hydrogen site location: inferred from neighbouring sites
*wR*(*F*^2^) = 0.124	H atoms treated by a mixture of independent and constrained refinement
*S* = 1.06	*w* = 1/[σ^2^(*F*_o_^2^) + (0.0643*P*)^2^ + 0.080*P*] where *P* = (*F*_o_^2^ + 2*F*_c_^2^)/3
3048 reflections	(Δ/σ)_max_ < 0.001
232 parameters	Δρ_max_ = 0.16 e Å^−^^3^
0 restraints	Δρ_min_ = −0.17 e Å^−^^3^

### Special details

**Table:**

Geometry. All s.u.'s (except the s.u. in the dihedral angle between two l.s. planes) are estimated using the full covariance matrix. The cell s.u.'s are taken into account individually in the estimation of s.u.'s in distances, angles and torsion angles; correlations between s.u.'s in cell parameters are only used when they are defined by crystal symmetry. An approximate (isotropic) treatment of cell s.u.'s is used for estimating s.u.'s involving l.s. planes.
Refinement. Refinement of *F*^2^ against ALL reflections. The weighted *R*–factor *wR* and goodness of fit *S* are based on *F*^2^, conventional *R*–factors *R* are based on *F*, with *F* set to zero for negative *F*^2^. The threshold expression of *F*^2^ > σ(*F*^2^) is used only for calculating *R*–factors(gt) *etc*. and is not relevant to the choice of reflections for refinement. *R*–factors based on *F*^2^ are statistically about twice as large as those based on *F*, and *R*–factors based on ALL data will be even larger.

### Fractional atomic coordinates and isotropic or equivalent isotropic displacement parameters (Å^2^)

**Table:**

	*x*	*y*	*z*	*U*_iso_*/*U*_eq_	
C10	0.4816 (3)	0.29498 (16)	0.43832 (11)	0.0499 (4)	
H10A	0.4176	0.3776	0.4244	0.060*	
H10B	0.5846	0.3166	0.4961	0.060*	
N4	1.1893 (4)	0.48736 (19)	0.26329 (15)	0.0742 (5)	
C8	0.1199 (3)	0.21672 (16)	0.52086 (10)	0.0429 (4)	
H8A	0.0453	0.2947	0.5041	0.052*	
H8B	−0.0076	0.1388	0.5218	0.052*	
C9	0.2779 (3)	0.18047 (15)	0.44755 (10)	0.0422 (4)	
H9A	0.3442	0.0987	0.4619	0.051*	
H9B	0.1793	0.1577	0.3889	0.051*	
N1	0.7649 (3)	0.08392 (17)	0.74199 (12)	0.0577 (4)	
O1	0.3165 (2)	0.03649 (10)	0.62896 (7)	0.0508 (3)	
N3	0.6215 (2)	0.25310 (13)	0.36539 (9)	0.0447 (3)	
N2	0.2451 (2)	0.25147 (13)	0.61277 (8)	0.0397 (3)	
C7	0.3267 (3)	0.15789 (14)	0.66164 (10)	0.0371 (3)	
C1	0.6397 (3)	0.16352 (15)	0.79517 (10)	0.0421 (4)	
C6	0.4305 (3)	0.20735 (14)	0.75834 (10)	0.0373 (3)	
C5	0.3172 (3)	0.29381 (15)	0.81381 (10)	0.0446 (4)	
H5	0.1789	0.3235	0.7893	0.053*	
C13	0.7777 (3)	0.16008 (16)	0.19449 (11)	0.0462 (4)	
H13	0.6336	0.1122	0.2092	0.055*	
O2	0.7750 (3)	0.46872 (11)	0.34395 (10)	0.0759 (5)	
C11	0.7537 (3)	0.34344 (15)	0.32101 (11)	0.0434 (4)	
C12	0.8745 (3)	0.28706 (15)	0.24415 (10)	0.0406 (4)	
C2	0.7255 (3)	0.20792 (18)	0.88686 (11)	0.0534 (4)	
H2	0.8642	0.1797	0.9123	0.064*	
C17	1.0877 (3)	0.36121 (17)	0.21943 (12)	0.0499 (4)	
C4	0.4052 (3)	0.33631 (18)	0.90419 (11)	0.0552 (4)	
H4	0.3274	0.3941	0.9404	0.066*	
C3	0.6096 (3)	0.2922 (2)	0.94020 (11)	0.0584 (5)	
H3	0.6696	0.3199	1.0013	0.070*	
C14	0.8889 (4)	0.10349 (19)	0.12450 (12)	0.0585 (5)	
H14	0.8207	0.0187	0.0920	0.070*	
C15	1.1026 (4)	0.1739 (2)	0.10312 (13)	0.0661 (5)	
H15	1.1816	0.1353	0.0569	0.079*	
C16	1.1994 (3)	0.2992 (2)	0.14894 (13)	0.0632 (5)	
H16	1.3437	0.3452	0.1331	0.076*	
H2N4	1.307 (5)	0.526 (3)	0.2455 (16)	0.090 (8)*	
H1N4	1.121 (4)	0.530 (2)	0.3056 (14)	0.067 (7)*	
H1N1	0.874 (4)	0.051 (2)	0.7690 (15)	0.075 (7)*	
H2N1	0.691 (4)	0.048 (2)	0.6872 (15)	0.068 (6)*	
H3N3	0.635 (3)	0.163 (2)	0.3542 (11)	0.056 (5)*	
H2N2	0.264 (3)	0.3384 (18)	0.6371 (11)	0.050 (5)*	

### Atomic displacement parameters (Å^2^)

**Table:**

	*U*^11^	*U*^22^	*U*^33^	*U*^12^	*U*^13^	*U*^23^
C10	0.0631 (10)	0.0392 (8)	0.0490 (9)	0.0105 (7)	0.0169 (8)	−0.0008 (7)
N4	0.0630 (11)	0.0602 (11)	0.0935 (14)	−0.0136 (9)	0.0212 (10)	0.0051 (10)
C8	0.0455 (9)	0.0428 (8)	0.0415 (8)	0.0113 (7)	0.0038 (7)	0.0034 (7)
C9	0.0510 (9)	0.0395 (8)	0.0362 (8)	0.0107 (7)	0.0028 (7)	0.0013 (6)
N1	0.0526 (9)	0.0666 (10)	0.0551 (10)	0.0235 (8)	0.0004 (8)	−0.0054 (8)
O1	0.0739 (8)	0.0280 (6)	0.0488 (6)	0.0120 (5)	−0.0041 (6)	−0.0009 (5)
N3	0.0601 (8)	0.0292 (7)	0.0478 (8)	0.0098 (6)	0.0173 (6)	0.0040 (6)
N2	0.0539 (8)	0.0288 (7)	0.0372 (7)	0.0100 (5)	0.0065 (6)	0.0007 (5)
C7	0.0422 (8)	0.0288 (7)	0.0402 (8)	0.0040 (6)	0.0072 (6)	0.0035 (6)
C1	0.0444 (8)	0.0385 (8)	0.0424 (8)	0.0010 (6)	0.0075 (7)	0.0054 (6)
C6	0.0434 (8)	0.0305 (7)	0.0372 (8)	0.0015 (6)	0.0067 (6)	0.0048 (6)
C5	0.0493 (9)	0.0413 (8)	0.0434 (9)	0.0065 (7)	0.0099 (7)	0.0033 (7)
C13	0.0529 (9)	0.0410 (9)	0.0457 (9)	0.0079 (7)	0.0072 (7)	0.0075 (7)
O2	0.0973 (10)	0.0261 (6)	0.1097 (11)	0.0086 (6)	0.0463 (9)	0.0033 (6)
C11	0.0495 (9)	0.0292 (8)	0.0534 (9)	0.0086 (6)	0.0085 (7)	0.0074 (6)
C12	0.0451 (8)	0.0365 (8)	0.0434 (8)	0.0102 (6)	0.0063 (7)	0.0133 (6)
C2	0.0526 (10)	0.0605 (11)	0.0450 (9)	0.0072 (8)	−0.0010 (8)	0.0050 (8)
C17	0.0474 (9)	0.0507 (10)	0.0534 (10)	0.0066 (7)	0.0048 (8)	0.0184 (8)
C4	0.0664 (11)	0.0561 (10)	0.0430 (9)	0.0097 (9)	0.0149 (8)	−0.0043 (8)
C3	0.0677 (12)	0.0676 (12)	0.0351 (8)	0.0025 (9)	0.0022 (8)	−0.0024 (8)
C14	0.0770 (13)	0.0562 (11)	0.0436 (9)	0.0165 (9)	0.0082 (9)	0.0017 (8)
C15	0.0751 (13)	0.0834 (15)	0.0485 (10)	0.0289 (11)	0.0214 (9)	0.0112 (10)
C16	0.0525 (11)	0.0830 (14)	0.0604 (11)	0.0139 (10)	0.0203 (9)	0.0233 (10)

### Geometric parameters (Å, º)

**Table:**

C10—N3	1.4548 (19)	C1—C2	1.394 (2)
C10—C9	1.507 (2)	C1—C6	1.401 (2)
C10—H10A	0.9700	C6—C5	1.391 (2)
C10—H10B	0.9700	C5—C4	1.376 (2)
N4—C17	1.359 (2)	C5—H5	0.9300
N4—H2N4	0.79 (3)	C13—C14	1.372 (2)
N4—H1N4	0.87 (2)	C13—C12	1.392 (2)
C8—N2	1.4502 (19)	C13—H13	0.9300
C8—C9	1.512 (2)	O2—C11	1.2306 (18)
C8—H8A	0.9700	C11—C12	1.480 (2)
C8—H8B	0.9700	C12—C17	1.406 (2)
C9—H9A	0.9700	C2—C3	1.368 (2)
C9—H9B	0.9700	C2—H2	0.9300
N1—C1	1.372 (2)	C17—C16	1.401 (2)
N1—H1N1	0.82 (2)	C4—C3	1.375 (3)
N1—H2N1	0.89 (2)	C4—H4	0.9300
O1—C7	1.2351 (17)	C3—H3	0.9300
N3—C11	1.3275 (19)	C14—C15	1.375 (3)
N3—H3N3	0.900 (18)	C14—H14	0.9300
N2—C7	1.3283 (18)	C15—C16	1.357 (3)
N2—H2N2	0.882 (17)	C15—H15	0.9300
C7—C6	1.492 (2)	C16—H16	0.9300
			
N3—C10—C9	110.24 (13)	C5—C6—C1	119.20 (14)
N3—C10—H10A	109.6	C5—C6—C7	120.55 (13)
C9—C10—H10A	109.6	C1—C6—C7	120.22 (13)
N3—C10—H10B	109.6	C4—C5—C6	121.39 (16)
C9—C10—H10B	109.6	C4—C5—H5	119.3
H10A—C10—H10B	108.1	C6—C5—H5	119.3
C17—N4—H2N4	118.3 (18)	C14—C13—C12	121.85 (16)
C17—N4—H1N4	122.2 (14)	C14—C13—H13	119.1
H2N4—N4—H1N4	119 (2)	C12—C13—H13	119.1
N2—C8—C9	114.52 (12)	O2—C11—N3	120.76 (15)
N2—C8—H8A	108.6	O2—C11—C12	121.93 (14)
C9—C8—H8A	108.6	N3—C11—C12	117.30 (13)
N2—C8—H8B	108.6	C13—C12—C17	118.79 (15)
C9—C8—H8B	108.6	C13—C12—C11	120.54 (13)
H8A—C8—H8B	107.6	C17—C12—C11	120.67 (14)
C10—C9—C8	113.67 (12)	C3—C2—C1	121.29 (16)
C10—C9—H9A	108.8	C3—C2—H2	119.4
C8—C9—H9A	108.8	C1—C2—H2	119.4
C10—C9—H9B	108.8	N4—C17—C16	120.07 (17)
C8—C9—H9B	108.8	N4—C17—C12	122.05 (17)
H9A—C9—H9B	107.7	C16—C17—C12	117.85 (16)
C1—N1—H1N1	116.9 (15)	C3—C4—C5	119.17 (16)
C1—N1—H2N1	116.2 (13)	C3—C4—H4	120.4
H1N1—N1—H2N1	123 (2)	C5—C4—H4	120.4
C11—N3—C10	122.79 (13)	C2—C3—C4	120.57 (16)
C11—N3—H3N3	117.6 (11)	C2—C3—H3	119.7
C10—N3—H3N3	119.2 (11)	C4—C3—H3	119.7
C7—N2—C8	122.81 (13)	C13—C14—C15	119.07 (18)
C7—N2—H2N2	119.3 (11)	C13—C14—H14	120.5
C8—N2—H2N2	117.9 (11)	C15—C14—H14	120.5
O1—C7—N2	121.85 (14)	C16—C15—C14	120.55 (17)
O1—C7—C6	121.82 (13)	C16—C15—H15	119.7
N2—C7—C6	116.33 (12)	C14—C15—H15	119.7
N1—C1—C2	120.17 (16)	C15—C16—C17	121.79 (17)
N1—C1—C6	121.40 (15)	C15—C16—H16	119.1
C2—C1—C6	118.37 (15)	C17—C16—H16	119.1
			
N3—C10—C9—C8	−178.84 (12)	C14—C13—C12—C11	−178.25 (14)
N2—C8—C9—C10	−59.28 (17)	O2—C11—C12—C13	−152.89 (16)
C9—C10—N3—C11	156.19 (15)	N3—C11—C12—C13	27.7 (2)
C9—C8—N2—C7	−71.30 (18)	O2—C11—C12—C17	26.6 (2)
C8—N2—C7—O1	5.5 (2)	N3—C11—C12—C17	−152.90 (15)
C8—N2—C7—C6	−174.30 (12)	N1—C1—C2—C3	177.18 (16)
N1—C1—C6—C5	−176.68 (14)	C6—C1—C2—C3	0.0 (2)
C2—C1—C6—C5	0.5 (2)	C13—C12—C17—N4	178.33 (16)
N1—C1—C6—C7	5.3 (2)	C11—C12—C17—N4	−1.1 (2)
C2—C1—C6—C7	−177.56 (13)	C13—C12—C17—C16	−3.5 (2)
O1—C7—C6—C5	−136.72 (15)	C11—C12—C17—C16	177.02 (14)
N2—C7—C6—C5	43.05 (18)	C6—C5—C4—C3	0.0 (2)
O1—C7—C6—C1	41.3 (2)	C1—C2—C3—C4	−0.5 (3)
N2—C7—C6—C1	−138.96 (14)	C5—C4—C3—C2	0.5 (3)
C1—C6—C5—C4	−0.5 (2)	C12—C13—C14—C15	0.3 (3)
C7—C6—C5—C4	177.54 (14)	C13—C14—C15—C16	−1.7 (3)
C10—N3—C11—O2	4.5 (3)	C14—C15—C16—C17	0.3 (3)
C10—N3—C11—C12	−176.08 (14)	N4—C17—C16—C15	−179.51 (18)
C14—C13—C12—C17	2.3 (2)	C12—C17—C16—C15	2.3 (3)

### Hydrogen-bond geometry (Å, º)

**Table:**

*D*—H···*A*	*D*—H	H···*A*	*D*···*A*	*D*—H···*A*
N4—H1*N*4···O2	0.87 (2)	2.10 (2)	2.711 (3)	126.6 (18)
N1—H2*N*1···O1	0.89 (2)	2.20 (2)	2.851 (2)	130.2 (19)
N3—H3*N*3···O1^i^	0.902 (19)	2.055 (19)	2.9286 (16)	162.8 (15)
N2—H2*N*2···O2^ii^	0.882 (17)	1.942 (18)	2.7872 (17)	160.3 (16)

Symmetry codes: (i) −*x*+1, −*y*, −*z*+1; (ii) −*x*+1, −*y*+1, −*z*+1.
